# The Cancer Antioxidant Regulation System in Therapeutic Resistance

**DOI:** 10.3390/antiox13070778

**Published:** 2024-06-27

**Authors:** Xuanhao Gu, Chunyang Mu, Rujia Zheng, Zhe Zhang, Qi Zhang, Tingbo Liang

**Affiliations:** 1 Department of Hepatobiliary and Pancreatic Surgery, The First Affiliated Hospital, Zhejiang University School of Medicine, Hangzhou 310003, China; xuanhao_gu@zju.edu.cn (X.G.); mu.chunyang@zju.edu.cn (C.M.); scuzz@zju.edu.cn (Z.Z.); 2 Zhejiang Provincial Key Laboratory of Pancreatic Disease, The First Affiliated Hospital, Zhejiang University School of Medicine, Hangzhou 310003, China; l210958@zju.edu.cn; 3 Zhejiang Clinical Research Center of Hepatobiliary and Pancreatic Diseases, Hangzhou 310003, China; 4 The Innovation Center for the Study of Pancreatic Diseases of Zhejiang Province, Hangzhou 310003, China; 5 Zhejiang University Cancer Center, Hangzhou 310003, China; 6 MOE Joint International Research Laboratory of Pancreatic Diseases, The First Affiliated Hospital, Zhejiang University School of Medicine, Hangzhou 310003, China

**Keywords:** antioxidant, reactive oxygen species, cancer therapy resistance, oxidative stress, redox signaling, NRF2

## Abstract

Antioxidants play a pivotal role in neutralizing reactive oxygen species (ROS), which are known to induce oxidative stress. In the context of cancer development, cancer cells adeptly maintain elevated levels of both ROS and antioxidants through a process termed “redox reprogramming”. This balance optimizes the proliferative influence of ROS while simultaneously reducing the potential for ROS to cause damage to the cell. In some cases, the adapted antioxidant machinery can hamper the efficacy of treatments for neoplastic diseases, representing a significant facet of the resistance mechanisms observed in cancer therapy. In this review, we outline the contribution of antioxidant systems to therapeutic resistance. We detail the fundamental constituents of these systems, encompassing the central regulatory mechanisms involving transcription factors (of particular importance is the KEAP1/NRF2 signaling axis), the molecular effectors of antioxidants, and the auxiliary systems responsible for NADPH generation. Furthermore, we present recent clinical trials based on targeted antioxidant systems for the treatment of cancer, assessing the potential as well as challenges of this strategy in cancer therapy. Additionally, we summarize the pressing issues in the field, with the aim of illuminating a path toward the emergence of novel anticancer therapeutic approaches by orchestrating redox signaling.

## 1. Introduction

**Figure 1:**
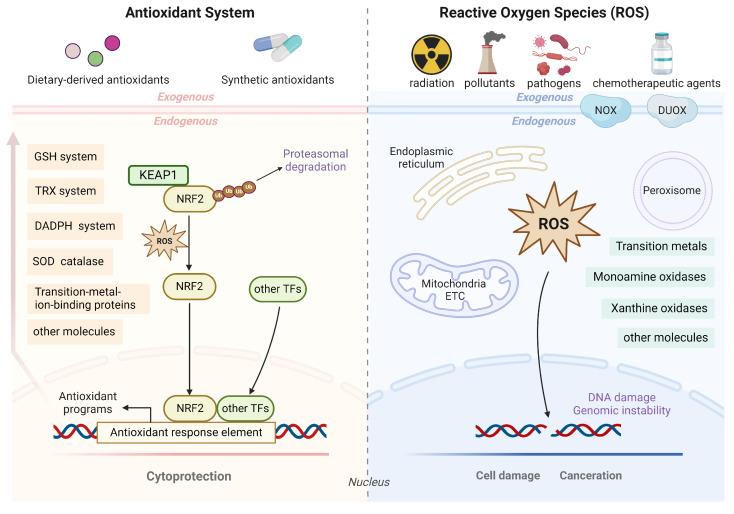
The composition of the antioxidant system and sources of ROS. The KEAP1-NRF2-ARE axis and other transcription factors are the core of the endogenous antioxidant system in cells, which includes numerous antioxidant effectors (such as the GSH/TRX system, superoxide dismutase, catalase) and auxiliary factors (the NADPH synthesis system) that work together with exogenous antioxidants to provide cellular protection under physiological regulation. The main sources of ROS within cells are the mitochondrial electron transport chain, endoplasmic reticulum, and peroxisome, involving many oxidases. External stimuli can also promote ROS production, leading to cell damage or cancer when in excess.

Antioxidants can be broadly defined as “substances that modulate redox signaling or/and decrease oxidative damage by reacting with oxidants” [1,2], such as reactive oxygen species (ROS), which is a collective term often used to refer to “unstable and reactive molecules that originate from oxygen during cellular metabolism” [3]. Cells can produce ROS through a variety of endogenous and exogenous mechanisms [4,5], and ROS include a wide range of types, including superoxide (O_2_·^−^), hydrogen peroxide (H_2_O_2_), and the hydroxyl radical (·OH). Under physiological conditions, different ROS can perform diverse functions, among which the most important is acting as signaling molecules to affect cellular behavior [4,6]. However, excessive ROS lead to oxidative stress and can exert toxic effects on DNA, proteins, lipids, and other biomolecules, ultimately leading to cell apoptosis or ferroptosis [7]. Hence, the maintenance of redox homeostasis, which is crucial for normal cellular physiological function, relies on intricate interactions between antioxidants and ROS and the precise regulation of their balance (Figure 1). The perturbation of this balance leads to the occurrence of many diseases, including cancer.

A substantial body of literature has demonstrated the pivotal roles of ROS in multiple facets of cancer development, including tumor initiation, progression, invasion, metastasis, microenvironment remodeling, and therapeutic response [3,7]. Metabolic abnormalities and oncogenic signals in cancer cells can increase ROS levels, thereby triggering adaptive redox responses to enhance antioxidant capacity and modify redox kinetics, which enables cells to maintain ROS below the toxicity threshold while still harnessing the role of ROS in promoting tumorigenesis [8,9]. Therefore, there is a prevailing misconception among the public that ROS are inherently potentially harmful, while antioxidants have positive effects on various aspects of human health, including cancer prevention and treatment [2]. In recent decades, there has been a lack of evidence supporting the protective effect of antioxidants against cancer [10]. Conversely, certain studies have indicated that supplementary antioxidants might actually contribute to the development of cancer [11,12]. Similarly, some randomized controlled clinical trials have consistently demonstrated that the addition of antioxidants did not enhance the efficacy of existing therapies and even resulted in unfavorable prognostic outcomes [13,14,15,16,17]. In fact, there are clinical recommendations advising against the concurrent use of antioxidant supplements during chemotherapy or radiotherapy [18,19]. A simplified explanation is that antioxidants can counteract the toxic effects of ROS on tumor cells caused by anticancer therapies such as chemotherapy and radiotherapy (which at least partially constitute the mechanisms of anticancer therapy), leading to the development of therapeutic resistance [20], which remains the principal impediment to a favorable prognosis for cancer patients [21]. In addition, KEAP1 (Kelch-like ECH-associated protein 1)/NRF2 (nuclear factor erythroid 2-related factor 2) signaling, recognized as one of the most important antioxidant regulators, plays a multifaceted role in the initiation and progression of some cancers [22,23]. This underscores the potential of KEAP1/NRF2 as a promising target for therapeutic intervention in some cases.

The involvement of ROS in the process of therapeutic resistance notwithstanding, a greater concern lies in antioxidant detoxifying systems, which play a crucial role in cancer cell survival upon exposure to anticancer therapies, and the deactivation of these defensive systems may enhance the efficacy of therapies, mitigate adverse effects, and optimize patients’ overall quality of life [8,24]. However, the complex nature of antioxidant detoxifying systems, which encompass a vast array of small molecules and enzymes, coupled with the intricacy involved in comprehending their regulation, presents a formidable challenge in regard to targeting the antioxidant capacity of tumor cells [1].

In this review, we summarize the literature to introduce the sources and composition of antioxidant systems, as well as the mechanisms related to tumor therapeutic resistance, and explore the clinical progress and future prospects of therapeutic interventions directly or indirectly targeting antioxidants.

## 2. Overview of Therapeutic Resistance in Cancer

**Figure 2:**
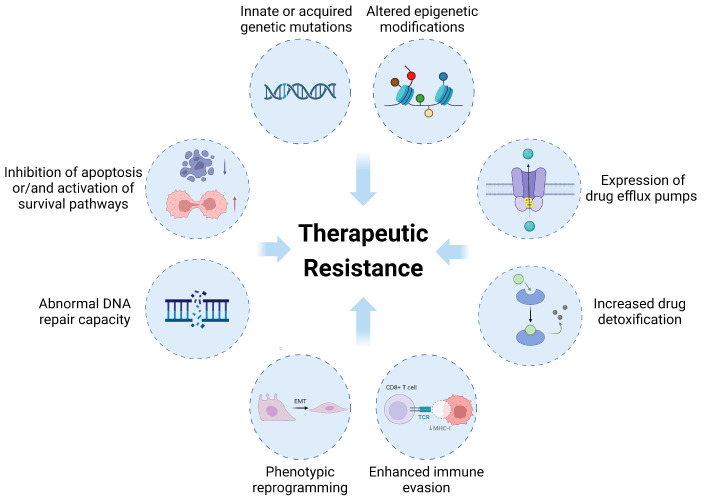
The mechanisms of resistance to antitumor therapies. In response to therapeutic interventions, cancer cells have the ability to undergo changes at various levels or interact with microenvironmental cells (most commonly impacting immune cells), which impedes optimal treatment outcomes. The red upward arrow in the figure indicates increased cell proliferation, and the blue downward arrow in the figure indicates decreased cell apoptosis.

The classical approaches to clinical cancer therapy include surgical resection, pharmacotherapy (including chemotherapy, targeted therapy, endocrine therapy, and immunotherapy), and radiotherapy. Among these modalities, pharmacotherapy has exhibited the most rapid advancements in research progress. Despite the approval of more than 200 therapeutic products for nearly 600 oncology indications since the turn of the 21st century, many patients still lack effective treatments, and innate or acquired drug resistance continues to limit clinical benefits even with the most advanced drugs [25,26]. Drug resistance can be mediated by the overactivation of detoxifying systems, e.g., glutathione-S-transferase (GST), which mediates the detoxification of reactive compounds using glutathione (GSH) [27], and glutathione peroxidase (GPX), which reduces H_2_O_2_ to water and lipid peroxides to alcohols, thus reining in the deleterious impacts of ROS [28]. This aspect, characterized by altered drug metabolism, is the focus of this review and will be further expounded upon. In addition, the drug resistance of cancer cells can be mediated by (1) innate (for the time being, undruggable) oncogenic mutations (e.g., *KRAS* G12D) or acquired target mutations (e.g., *EGFR* T790M, *BCR-ABL* E255K) [29,30]; (2) the promotion of drug efflux through the overexpression of ATP-binding cassette (ABC) transporters (e.g., multidrug resistance protein 1 (MDR1), multidrug resistance-associated protein 1 (MRP1)) [31]; (3) the inhibition of cell death or/and the activation of survival pathways (e.g., S-glutathionylation of caspase-3, activation of Bcl-2/Bcl-xl) [32,33]; (4) an increased capacity for DNA damage repair (e.g., poly (ADP-ribose) polymerase-1 (PARP-1), apurinic/apyrimidinic endonuclease (APE-1)) [34,35]; (5) the formation of cancer stem cells (CSCs) and the remodeling of phenotypes (e.g., epithelial-to-mesenchymal transition (EMT)) [3,36]; and (6) enhanced immune evasion [37] (Figure 2). More details can be found in several excellent reviews [21,38]. Currently, there is an imperative to devise novel drug utilization tactics for overcoming drug resistance.

With respect to the resistance of cancer cells to radiotherapy, in addition to not involving drug metabolism, other aspects are similar to drug resistance, including radiation-induced gene mutation or abnormal expression, DNA damage repair, cell cycle arrest, ROS scavenging, escape from apoptosis, enhanced autophagy, modification of the tumor environment (TME), and metabolic reprogramming [39,40]. The mechanisms involved in antioxidant systems undoubtedly play important roles, which will be discussed below.

## 3. Antioxidant Defense System and Antioxidant-modulated Therapeutic Resistance

From the perspective of sources, antioxidants can be divided into two categories: exogenous and endogenous. Exogenous antioxidants are mainly obtained through dietary intake, and some synthetic antioxidants are also used in specific medical applications [41]. Dietary-derived antioxidants, including vitamin E and other phenolic compounds (e.g., flavonoids, anthocyanins, caffeic acid), vitamin A (retinal), vitamin C (ascorbate), and carotenoids, some of which are essential to humans [42,43,44], are frequently nutrient molecules that also play crucial roles in antioxidant activity. The synthetic antioxidants that have been used in clinical practice include N-acetylcysteine (NAC), ebselen, and edaravone [41]. Endogenous antioxidants synthesized in vivo constitute the primary components of the antioxidant defense system and can be classified into two categories: enzymatic and nonenzymatic. The former include (1) the enzymes involved in glutathione (GSH) synthesis (e.g., glutamate–cysteine ligase (GCL), GSH synthetase (GSS)); (2) GSH-dependent ROS-scavenging enzymes (e.g., peroxiredoxin (PRDX), thioredoxin reductase (TrxR)); (4) the enzymes involved in NADPH production (e.g., glucose-6-phosphate dehydrogenase (G6PD), isocitrate dehydrogenase (IDH)); (5) other enzymes, such as catalase (CAT) and superoxide dismutase (SOD). The latter comprise (1) antioxidant transcription factors (e.g., NRF2, activator protein-1 (AP-1), nuclear factor kappa B (NF-κB)) [45]; (2) reduced GSH and NADPH; (3) proteins binding transition metal ions (e.g., albumin, haptoglobin, transferrin, caeruloplasmin) [46]; (4) trace elements (e.g., selenium, zinc) [47,48]; (5) other molecules, such as ubiquinone (CoQ), plasmalogen, and melatonin [41,49]. As commonly acknowledged, transcription factors such as NRF2 play a key role in the antioxidant defense system and can be activated by ROS, leading to the enhanced transcription of antioxidant gene targets, including enzymes involved in GSH synthesis, utilization, and regeneration, NADPH generation, and thioredoxin-related enzymes, as enumerated above, thus precisely governing the redox homeostasis of cells [7,45]. In this section, the focus will primarily be on ROS-responsive transcription factors, the GSH system, the TRX system, and NADPH synthesis to investigate the mechanisms of antioxidants in resistance to cancer therapy.

### 3.1. Antioxidant Transcription Factor Network in Cancer Therapeutic Resistance

#### 3.1.1. KEAP1–NRF2–ARE Axis in Cancer Therapeutic Resistance

**Figure 3:**
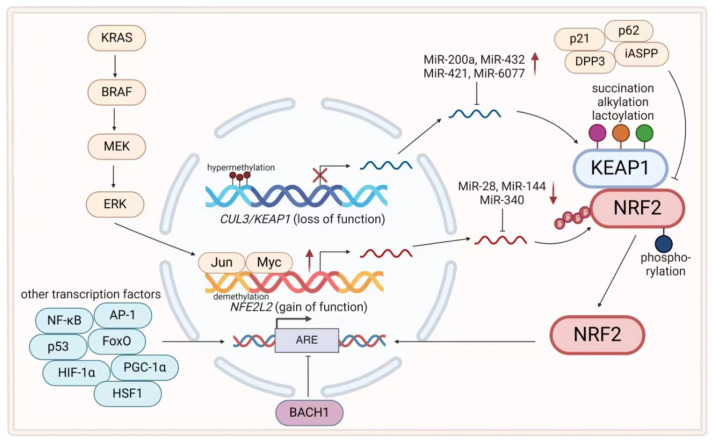
The antioxidant transcription factor network in cancer therapeutic resistance. In a variety of cancers, the KEAP1-NRF2-ARE axis is often altered at different levels: (1) mutations at the genomic level; (2) regulation of transcription by epigenetic factors and oncogenic proteins; (3) regulation of mRNA stability by noncoding RNAs; (4) posttranslational modification of NRF2/KEAP1 protein by different types of metabolites and regulatory proteins; (5) multiple protein chaperones interacting with NRF2/KEAP1. In addition, other transcription factors also form an important part of the core regulatory network. These changes enhance the ability of cancer cells to cope with oxidative stress and thus survive various antitumor treatments. The right up and down arrows in figure mean increasing or decreasing miRNA expression in tumor cells; the X signal means reducing transcription of *CUL3/KEAP1* gene.

Nuclear factor erythroid 2-related factor 2 (NRF2) is a basic-region leucine zipper (bZIP) transcription factor composed of seven Nrf2-ECH homology domains. Under unstressed conditions, most NRF2 is repressed in the cytoplasm by Kelch-like ECH-associated protein 1 (KEAP1), which is a substrate adaptor protein of the cullin 3 (Cul3)-containing E3 ubiquitin ligase that promotes NRF2 degradation via the proteasome. Elevated levels of ROS trigger the dissociation of NRF2 from KEAP1, its translocation to the nucleus, and the subsequent formation of heterodimers with the musculoaponeurotic fibrosarcoma (MAF) protein to bind AREs (gene sequences called antioxidant response elements). This leads to the transactivation of more than 200 genes, thereby enhancing the antioxidant capacity of the cells [50,51]. Dysregulation of this axis is involved in all stages of cancer and, of course, in resistance to therapy [51]. There were four main alterations at various levels (Table 1) (Figure 3).

***Alterations at the Genetic Level.***

Initially discovered in lung cancer [52], gain-of-function mutations in *NFE2L2* (the gene encoding NRF2) or loss-of-function mutations in *KEAP1* were found to be frequently acquired by cancers [53], with a frequency of about 20% in lung adenocarcinoma (LUAD) [54]. In general, the literature regarding mutations in *KEAP1* as a tumor suppressor gene appears to be more extensive than that concerning *NFE2L2*. The earliest report on drug resistance was conducted by Tsutomu Ohta et al., who reported that *KEAP1* mutations located within the IVR and DGR domains occurred in a subset of Japanese lung cancer patients (3/29, 10%), leading to the attenuated inhibition of KEAP1 and the enhanced transcriptional activity of NRF2, giving rise to growth advantages and cisplatin resistance in cancer cells [55]. Similar mutations occurred in 5-fluorouracil (5-FU)-resistant gallbladder cancer (4/13, 31%) and platinum-based chemotherapy-resistant ovarian cancer (4/14, 29%), both of which could be sensitized by NRF2 siRNA in vitro [62,63]. Chemoresistance mediated by *KEAP1* mutations has also been observed in prostate cancer, melanoma, and head and neck squamous cell carcinoma (HNSCC), indicating poor treatment response [64,65,66]. Recently, the resistance mechanisms mediated by *KEAP1* mutations have been increasingly revealed in other therapeutic approaches, especially in lung cancer. For example, in radiation-resistant KEAP1-deficient lung cancer cells, the ubiquinone (CoQ)–ferroptosis suppressor protein 1 (FSP1) axis was activated to hinder radiation-induced ferroptosis [57], and increased glutathione levels and DNA damage repair capacity might also partially explain this phenomenon [58]. *KEAP1* mutations, together with mutant *KRAS*, conferred immunotherapy resistance to LUAD patients, possibly by damaging immune cell infiltration or suppressing CD103 dendritic cell-mediated CD_8_^+^ T-cell immunity, which could be rescued by the glutaminase inhibitor CB-839 [59,60]. The protein EMSY was stabilized in *KEAP1*-depleted cells to impair the interferon response, revealing the role of *KEAP1* mutations in immune evasion [61]. In targeted therapy, R16, a small-molecule agent, bound to mutant KEAP1 and reversed its ability to inhibit NRF2, thereby sensitizing lung cancer cells to gefitinib [56]. Although *NFE2L2* mutations are relatively rare, it has been reported that DLG motif mutations in NRF2 in hepatocellular carcinoma (HCC) and NRF2-mutant HNSCC cell lines upregulated NRF2 transcriptional activity and might promote chemoradiation resistance [67,68]. In addition, NRF2 overexpression in esophageal squamous cell carcinoma (ESCC) due to *NFE2L2* amplification was also associated with poor prognosis, as indicated by a change in gene copy number [69].

***Alterations at the Transcriptional and Translational Levels.***

The activation of oncogenic proteins (e.g., K−Ras^G12D^, B−Raf^V619E^, and Myc^ERT2^) is a hallmark of cancer in which intracellular ROS levels are inhibited by increased NRF2 transcription [70], thereby providing cytoprotection and possibly being associated with a poor response to initial therapy in some patients. In pancreatic cancer, the activation of the KRAS/ERK pathway upregulated protein interacting with never in mitosis A1 (PIN1), which synergized with c-Myc and NRF2 to maintain redox balance and promote cell survival [71]. The overexpression of NRF2 could also increase the risk of cytarabine resistance in a ROS-independent manner by inhibiting MutS Homolog 2 (MSH2) expression in acute myeloid leukemia (AML) [72].

The epigenetic regulation of *KEAP1/NEF2L2* gene expression has garnered increasing attention in recent years, and the major mechanisms involved include DNA methylation, miRNAs, and histone modifications. The hypermethylation of CpG islands catalyzed by DNA methyltransferases (DNMTs) in the promoters of *KEAP1* inhibited its transcriptional activity and led to the accumulation of NRF2, which conferred growth advantages and therapeutic resistance to many cancers, such as glioma, colorectal cancer (CRC), and lung cancer [73,74,75]. Additionally, it has been reported that the transcription factor stimulating protein-1 (SP1) exhibited impaired binding to the promoter of *KEAP1* in A549 lung cancer cells compared to normal bronchial epithelial cells, potentially attributed to the hypermethylation of the *KEAP1* promoter [76]. Conversely, a demethylation status of the *NEF2L2* promoter was found in colorectal tumor samples [77]. Relatively limited literature exists on histone modifications, with one notable example being the capacity of NRF2 to facilitate rapid cell proliferation in EZH2 (enhancer of zeste homolog 2)-deficient lung cancer cells, which potentially contributes to therapeutic resistance. This was due to the epigenetic silencing effect of EZH2, a crucial component of the polycomb repressive complex 2 (PRC2) that functioned as a histone H3K27 trimethyltransferase, on *NEF2L2* transcription [78].

Early studies on miRNAs revealed that the abundance of miR-144 was lower in 5-fluorouracil-resistant HCC and that the abundance of miR-144-3p was lower in cisplatin-resistant lung cancer cells [79,80], while transfection with miR-144 mimics or the re-expression of NRF2 reversed this resistance phenotype. This was because miR-144 can degrade mRNA by directly binding to the 3′-untranslated region (3′-UTR) of NRF2 mRNA, thereby reducing the translation of NRF2. Conversely, some miRNAs, such as miR-200a in breast cancer, miR-432 in ESCC, and miR-421/miR-6077 in lung cancer, exhibit carcinogenic effects by negatively regulating KEAP1 mRNA levels, leading to resistance to different chemotherapeutic agents [81,82,83,84]. As reported by Bingjie Liu et al., NRF2 could also reversely regulate miRNA by increasing the transcription of miR-196a to decrease the expression of glycolipid transfer protein (GLTP), which caused resistance in lung cancer cells to gefitinib and other tyrosine kinase inhibitors (TKIs) [85]. However, few studies have investigated the regulatory role of miRNAs in immunotherapy and radiotherapy resistance mediated by the KEAP1-NRF2-ARE axis.

***Alterations at the Posttranslational Modification Level.***

KEAP1-dependent NRF2 ubiquitination is known to be the major NRF2 ubiquitination pathway. Biologically, the cysteine-rich IVR domain and other Cys-containing domains (Kelch, BTB) of KEAP1 are broad-spectrum electrophile sensors that can be modified by oxidized/electrophilic substances to regulate NRF2 ubiquitination [98], so the oxidation level of thiol groups in KEAP1 directly reflects the activity of NRF2. In addition, cysteine residues can also be modified by metabolites in tumor cells [99]. For example, in tumor cells with fumarate hydratase (FH) deficiency, KEAP1 succination is induced by the abnormal accumulation of fumarate (which is weakly electrophilic), which, in turn, stabilizes NRF2 and activates the antioxidant pathway. This phenomenon is particularly common in papillary renal cell carcinoma type 2 (pRCC-2) and might be associated with a poor response to therapy and poor prognosis [86]. In addition, itaconate, which originates from the TCA cycle metabolite cis-aconitate and is catalyzed by aconitate decarboxylase 1 (ACOD1), can alkylate KEAP1, resulting in NRF2 activation in macrophages [87]. However, the underlying mechanism of itaconate in cancer therapeutic resistance remains unclear. A recent high-throughput screening revealed that the pyruvate kinase inhibitor sAKZ692 caused the accumulation of the glycolysis metabolite glyceraldehyde 3-phosphate (G3P), which led to the lactoylation of KEAP1 and NRF2-dependent transcriptional activation [88]. ROS generated during treatment can inhibit pyruvate kinase [3], leading to G3P accumulation, which might partially explain the occurrence of acquired therapeutic tolerance. More special modifications of KEAP/NRF2 by cell metabolites are worth exploring. Furthermore, the phosphorylation of NRF2 serine/threonine/tyrosine residues by protein kinases such as MAPK, PKC, ERK, GSK-3, and PI3K is a frequently observed posttranslational modification that has been reviewed by Tian Liu et al. [89].

***Alterations at the Protein Interaction Level.***

The cross-talk between KEAP1/NRF2 and several proteins has been identified. The p21 (CDKN1A) protein is a cyclin-dependent kinase inhibitor (CDKI) that competes with KEAP1 for binding to the DLG and ETGE domains of NRF2, thereby reducing NRF2 degradation and enhancing the antioxidant capacity of cells [90]. The NRF2 protein can also directly bind to the *P21* promoter, thereby establishing a positive feedback loop that activates *P21* expression and facilitates A549 cell survival under oxidative stress induced by H_2_O_2_ [91]. However, other findings suggested that the decreased expression or excessive degradation of p21 results in radioresistance in lung cancer cells, whereas the upregulation of p21 strengthens the radiosensitivity of NSCLC cell lines [100,101]. The p62 protein (sequestosome 1, SQSTM1), which is known as a selective substrate for autophagy to degrade protein aggregates, directly binds to KEAP1 via its STGE domain, which is identical to the ETGE domain of NRF2. Subsequently, p62 sequesters KEAP1 in autophagosomes, thereby reducing NRF2 ubiquitination, increasing NRF2 stability, and activating antioxidant/detoxifying genes such as *NAD(P)H quinone dehydrogenase 1 (NQO1)* and *heme oxygenase 1 (HMOX1)* to protect HCC cells from ferroptosis induced by erastin and sorafenib [92,93]. Dipeptidyl peptidase 3 (DPP3) also competes with NRF2 for KEAP1 in a similar manner, and a recent report demonstrated its high expression in ESCC cells; moreover, the knockdown of DPP3 rendered cells more susceptible to oxidative stress induced by cisplatin or paclitaxel [94,95]. Moreover, genetic ablation of the inhibitor of apoptosis stimulating protein of p53 (iASPP)/NRF2/macrophage-colony stimulating factor (M-CSF) axis suppressed the growth of doxorubicin-resistant colon cancer xenografts, indicating that the interaction between iASPP and KEAP1 was responsible for mediating chemotherapy resistance [96]. Notably, several other protein chaperones, including APC membrane recruitment protein 1 (AMER1), partner and localizer of BRCA2 (PALB2), and BRCA1, have been identified as disruptors of the KEAP1-NRF2 interaction, highlighting the intricate regulation of NRF2 [94,97].

#### 3.1.2. Other Antioxidant Transcription Factors in Cancer Therapeutic Resistance

Although NRF2 plays an important role, other constituents of the antioxidant transcription factor network are also indispensable for maintaining cellular redox homeostasis and are likely to be regulated by ROS at varying threshold levels [7] (Figure 3). The hierarchical oxidative stress response theory was proposed in relevant studies as early as 20 years ago. Specifically, NRF2 plays a role in the primary defense against low levels of ROS, while transcription factors such as NF-κB and AP-1 play a crucial role in the secondary defense by activating a cascade of inflammatory and antioxidant pathways to combat increased ROS. In instances where oxidative stress becomes overwhelming, a third-level defense mechanism, namely, apoptosis, is initiated [102]. However, the precise regulatory mechanism by which ROS modulate specific transcription factors remains unclear.

The most important tumor suppressor, p53, regulates cellular function by modulating multiple target genes so that cells can cope with stress or preventing alterations that lead to genomic instability. The crucial role of p53 in the induction of various antioxidant enzymes (such as GPX, GRX3, SOD2, and CAT) and NRF2 has been convincingly demonstrated [103]. However, p53 also has detrimental effects on tumor therapy due to the presence of multiple resistance mechanisms, particularly two pathways associated with antioxidant defense—the p53/immediate-early response 5 (IER5)/heat shock factor 1 (HSF1) axis and the p53/p21/NRF2 axis [104].

Although the main role of NF-κB is to stimulate inflammation and the immune response by inducing cytokines, chemokines, and adhesion molecules and subsequently eradicating invading pathogens and external stimuli, it also regulates the expression of antioxidant genes and participates in therapeutic resistance, involving enzymes that scavenge ROS (SOD1/2, GPX, CAT), synthesize GSH (GCLC/GCLM), produce NADPH (G6PD), and detoxify xenobiotics (NQO1, GST1) [105,106]. In lung cancer cells, NF-κB activated by mutant p53 or KRAS protected cells from cisplatin- or paclitaxel-induced apoptosis, while the NF-κB inhibitor PS1145 suppressed the growth of resistant cells [107]. A similar phenomenon has been observed in pancreatic cancer [108]. In recent studies, the NF-κB signaling pathway was implicated in the genesis of cancer stem cells, a pivotal attribute associated with cancer drug resistance [109]. Therefore, targeting the NF-κB pathway is expected to be a new strategy for reversing cancer drug resistance clinically [110]. However, paradoxically, NF-κB can also transactivate the expression of some pro-oxidative genes, such as *PTGS2* (encoding COX2) and *NOS2*, thereby attenuating the significance of antioxidant resistance mechanisms [105].

Activator protein (AP-1) is a dimeric transcription complex family composed of diverse protein subunits (Jun, Fos, ATF, and the MAF family), which are ubiquitously present within cells and play a critical role in regulating various physiological and pathological processes, including counteracting damage induced by environmental stimuli such as oxidative stress through CAT, SOD1, GST1, and other proteins [111,112]. Recent research has shown that diclofenac can induce intracellular ROS production by inhibiting the activity of AP-1 and NF-κB, thereby enhancing the sensitivity of cisplatin-resistant gastric carcinoma (GC) cells [113]. Moreover, the AP-1 family members JunB/FOSL2, which are activated by chronic exposure to H_2_O_2_, increase catalase expression and may endow MCF7 breast cancer cells with resistance to cisplatin [114].

The forkhead box, class O (FOXO) family comprises crucial transcription factors that modulate the cellular stress response and facilitate antioxidant activity. It can be dynamically regulated by multiple levels of ROS, thereby activating the expression of diverse antioxidant molecules, including SOD2, CAT, PRDX3/5, and ceruloplasmin [115]. A positive correlation has been identified between PTP nonreceptor type 12 (PTPN12) and FOXO1/3 in human breast cancer tissues. In vitro, the loss of PTPN12 resulted in excessive phosphorylation of pyruvate dehydrogenase kinase 1 (PDK1) and thus inhibited FOXO1/3, which increased intracellular ROS levels and suppressed normal growth. However, cells containing PTPN12 grew well under conditions of antioxidant depletion (GSH or NADPH deprivation) [116]. This finding implies that tumors have the potential to respond to external stimuli via FOXO regulatory mechanisms.

BTB domain and CNC homolog 1 (BACH1), in contrast to the aforementioned transcription factors, primarily functions as a regulator of heme and iron metabolism by suppressing the transcription of protective genes such as *FTH1*, *FTL1*, *HMOX1*, and *SLC40A1*. It also inhibits *TrxR1* and key genes involved in GSH synthesis, such as *GCLC/GCLM* and *SLC7A11* [117]. In BRAF^V600E^ MeOV-1 melanoma cells, vemurafenib, a BRAF^V600E^ inhibitor, showed limited efficacy because the expression of BACH1 was reduced, and HO-1 (encoded by *HOMX1*) was subsequently upregulated. HO-1 promoted the immune escape of melanoma cells, while the HO-1 inhibitor tin mesoporphyrin IX synergized with vemurafenib to effectively eradicate the tumor [118]. This suggests that inhibiting HO-1 may have a potential role in treatment.

Additionally, three other regulatory factors, PPAR-gamma coactivator 1α (PGC-1α), hypoxia-inducible factor 1 (HIF-1), and HSF1, are also implicated in antioxidant regulation through distinct mechanisms. Their contributions to resistance to cancer therapy can be inferred from the studies in [119,120,121] and will not be elaborated upon here. In summary, the regulation of the antioxidant transcription factor network is highly intricate and may also manifest in the resistance to various therapies observed among different types of cancer.

### 3.2. The GSH Antioxidant System in Cancer Therapeutic Resistance

**Figure 4:**
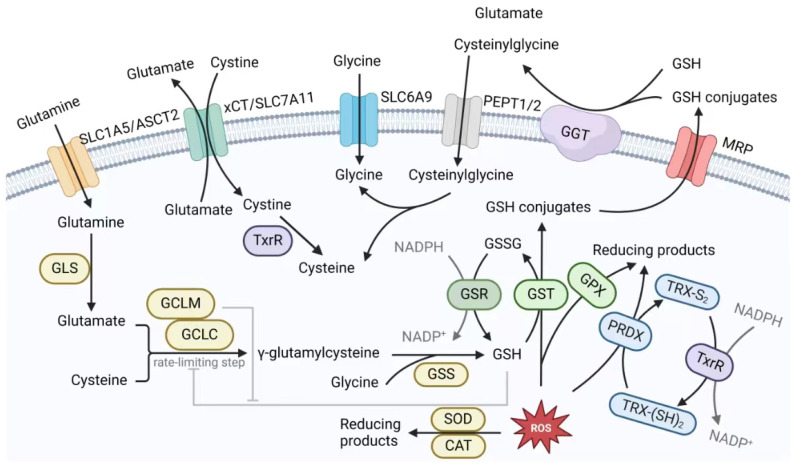
The GSH and TRX antioxidant system in cancer therapeutic resistance. The GSH system includes synthesis and utilization systems: the former includes enzymes directly catalyzing condensation reactions, such as GCL and GSS, and amino acid transporters, such as SLC1A5, SLC7A11, and SLC6A9; the latter includes the important detoxifying enzymes GST, GGT, and GPX. GSR is the catalytic enzyme responsible for the regeneration of GSH from GSSG in cells. The TRX system includes TRX, TrxR, PRDX, and TXNIP. Both systems depend on reduced equivalent NADPH for proper functioning.

Glutathione (GSH) is an effective antioxidant that widely exists in eukaryotic cells in two forms: thiol-reduced (GSH) and disulfide-oxidized (GSSG) forms. As a free radical scavenger and detoxifier, GSH plays an important role in cell division, proliferation, and differentiation and is one of the most commonly elevated metabolites under oxidative stress conditions such as drug stimulation [122]. In recent years, mounting evidence has revealed the necessary role of GSH in tumor initiation, progression, and metastasis and the acquisition of therapeutic resistance. Consequently, it has been proposed that enhancing the effect of anticancer therapy could be achieved through GSH depletion and the augmentation of ROS levels [123,124]. In this section, we will describe the mechanisms of therapeutic resistance from the perspectives of GSH synthesis and salvage (Figure 4).

#### 3.2.1. The GSH Synthesis System in Cancer Therapeutic Resistance

***Enzymes Associated with the De Novo Synthesis of GSH.***

The synthesis of GSH necessitates the utilization of glutamate, cysteine, and glycine as substrates and is achieved through a two-step ATP-dependent enzymatic reaction. The first step is catalyzed by glutamate–cysteine ligase (GCL), which is composed of a catalytic subunit (GCLC) and a modifier subunit (GCLM), in which glutamate and cysteine are linked by a γ-peptide bond to form γ-glutamylcysteine [122]. This is the rate-limiting step in GSH synthesis, so the enzymatic activity of GCL is critical for maintaining intracellular GSH levels. The *GCLC/GCLM* gene can be transactivated by NRF2 and NF-κB under conditions of oxidative stress, resulting in an increase in the cytoplasmic GCLC/GCLM ratio [125]. Increasing evidence in recent years has suggested that GCLC is the crux of cancer therapeutic resistance. High GCLC levels were found in HCC tumor tissues and were associated with poor overall survival (OS) and disease-free survival (DFS) in patients after curative treatment [126], while MYC-driven inhibition of GCLC through miR-18a increased the sensitivity of liver cancer cells to oxidative stress [127]. In pancreatic ductal adenocarcinoma (PDAC), the inhibition of GCLC expression and impairment of GSH biosynthesis caused by hyperglycemia significantly increased the sensitivity of patient-derived xenograft models to FOLFIRINOX chemotherapy compared to normal blood glucose [128]. In HNSCC, GCLC can also be affected by the histone methyltransferase G9a to promote cisplatin resistance [129]. Through pancancer single-cell RNA sequencing, *GCLC* was found to be among the top-ranked genes associated with cancer stemness and was found to be strongly related to immune checkpoint inhibitor (ICI) resistance in melanoma and basal cell carcinoma [130]. Moreover, the expression levels of GCLC could also serve as a predictive indicator for the radiosensitivity of HCC [131]. Since GCLM merely increases the threshold for GSH’s negative feedback inhibition without catalyzing the initial synthesis step [122], it may not be important in therapeutic resistance.

The second step of GSH synthesis is catalyzed by GSH synthetase (GSS), which adds glycine to γ-glutamylcysteine. GSS is not a rate-limiting enzyme and is not subject to negative feedback regulation by GSH. Although studies have reported that single-nucleotide polymorphisms (SNPs) of GSS are associated with recurrence after transurethral resection and poor response to intravesical BCG instillation in nonmuscle invasive bladder cancer (NMIBC) [132], conflicting results have shown that decreased GSH synthesis due to low GCLC/GSS expression contributes to erlotinib resistance in EGFRT790M NSCLC [133]. The current findings indicate that the underlying mechanisms by which GSS confers resistance to cancer therapy remain elusive and warrant further investigation. In addition, glutaminase 1/2 (GLS1/2) decomposes glutamine in the cytoplasm to provide glutamate for GSH synthesis. Studies have shown that GLS1 mediates resistance to trastuzumab in HER-2-positive gastric cancer by promoting M2 macrophage polarization [134]. In prostate cancer treated with androgen deprivation therapy, suppressed GLS1 expression compensated for the expression of the isozyme glutaminase C, which limited therapeutic efficacy [135].

***Transporters and Membrane Enzymes.***

Cysteine is considered the most important amino acid for combating oxidative stress [2]. In normal cells, it can be recycled through protein degradation or synthesized via de novo synthesis (the transsulfuration pathway). However, cancer cells mainly take up cysteine (in the oxidized form, cystine) from the extracellular environment due to their substantial need to adapt to high levels of ROS. Thus, the cystine/glutamate antitransporter xCT (encoded by the gene *SLC7A11* and subunit gene *SLC3A2*) is highly expressed on the cell membrane in many cancer types [2,136]. It can also be inhibited by sulfasalazine and erastin, the two most common inhibitors, and thus significantly alleviates drug resistance in many cancers, such as CRC and CD-133-positive HCC [137,138,139]. In recent years, an increasing number of key factors have been shown to regulate xCT, highlighting the crucial role of this transporter in maintaining cellular redox homeostasis. For example, xCT is upregulated by the transcription factor SOX2 in lung cancer stem-like cells (CSLCs) and causes resistance to ferroptosis induced by erastin [140]. An anti-xCT tumor vaccine significantly enhanced the efficacy of HER2 antibodies by inducing cytotoxic T cells and affecting CSC viability and self-renewal [141]. In contrast, a recent study revealed that, in some cancers, differentiated cells (those with enhanced stemness) were more sensitive to ferroptosis, as they had more xCT upregulated by the deubiquitinase DUBA than undifferentiated cells [142]. In addition to ferroptosis, xCT inhibition selectively induced apoptosis in CD44v-expressing EGFR-targeted-therapy-resistant HNSCC cells without affecting CD44v-negative cells, where CD44v was thought to stabilize xCT to promote cystine uptake, and the same result was shown in 5-fluorouracil-resistant gastric cancer [143,144]. In *EGFR*-mutant lung cancers, targeting aldo-keto reductase family 1 member B1 (AKR1B1), a key upstream regulatory protein of xCT that is upregulated in all resistant cell models, overcomes resistance to several TKIs by blocking cystine uptake and GSH accumulation [145]. Similarly, in ovarian cancer cells, xCT expression is significantly greater than that in normal tissues and is regulated by many cellular modulators, such as SNAI2, ARID1A, HRD1, and several noncoding RNAs [146], which was also reviewed by Jinyun Liu et al. [147], suggesting a number of potential targets for overcoming chemotherapy resistance. Furthermore, sulfasalazine sensitized aggressive glioblastoma (GBM) to γ-knife radiosurgery and reduced the rate of recurrence, which could be reversed by the antioxidant N-acetylcysteine NAC [148]. xCT also reduced radiosensitivity in a similar protective manner in ESCC [149]. Sulfasalazine has been utilized in clinical trials due to its numerous advantages demonstrated in laboratory studies, which will be described below.

As previously mentioned, xCT plays a crucial role in the uptake of cystine by tumor cells; however, it also has biological limitations. In other words, for each molecule of cystine to be absorbed, a molecule of glutamate must be expelled from cells. Additionally, NADPH is consumed during the reduction of cystine by TxrR1 within cells [2,122]. Therefore, the direct decomposition of GSH provides a more cost-effective approach for obtaining cysteine and is perhaps a preferable method for tumor cells. The membrane-bound hydrolase γ-glutamyl transferase (GGT), a clinically important biomarker of hepatobiliary diseases that is highly expressed in many cancers, degrades extracellular GSH to glutamate and cysteinylglycine, which are transported into the cell by the oligopeptide transporter (PEPT) 1/2 transporter and then decomposed into cysteine and glycine via a process catalyzed by dipeptidase [2,150]. The role of GGT in mediating cisplatin resistance in HeLa cells and renal tubular cells has been well established. Specifically, cisplatin readily forms adducts with cysteinylglycine, which cannot be transported across the plasma membrane. In addition, this reaction occurs at a rate 10 times faster than that of GSH due to the greater affinity of the -SH group in cysteinylglycine, thereby reducing the generation of ROS and alleviating the cytotoxicity of cisplatin [151,152]. Similar adducts were also detected in the plasma of patients treated with oxaliplatin [153]. In addition, not surprisingly, the KEAP1/NRF2 axis is also involved in the regulation of GGT in cisplatin resistance [154]. These findings imply that GGT has the potential to serve as a promising prognostic indicator for platinum sensitivity in patients. Recently, a supramolecular Pt(iv) prodrug nanoassembly delivery system containing the GGT inhibitor OU749 or NBDHEX was shown to maximize the therapeutic effect of platinum drugs in A549 cells by disrupting the redox balance [155,156]. In addition, the combination of GGT inhibitors and ferroptosis inducers is also a therapeutic strategy because glioblastoma cells expressing GGT are resistant to ferroptosis induced by cystine deprivation [157]. However, the role of GGT in radiotherapy, targeted therapy, and immunotherapy has not been fully elucidated.

Another transporter, solute carrier family 1, member 5 (encoded by *SLC1A5/ASCT2*), the main pathway for glutamine entry into cells [122], is induced by HIF-2α under hypoxic conditions to promote glutamate-dependent ATP production and GSH synthesis, which renders pancreatic cancer cells resistant to gemcitabine [158], and the amphireguli/MEK/ERK/NRF2 signaling pathway can positively upregulate SLC1A5 to support cisplatin resistance in chondrosarcoma [159]. SLC1A5, whose ablation sensitized tumors to the mTORC1 inhibitor rapamycin, was also essential for metabolic reprogramming and the rapid proliferation of *KRAS*-mutant colorectal cancer [160]. In drug-resistant NSCLC, apatinib inhibited GLS1, forcing cancer cells to initiate the amino acid response (AAR), which induced SLC1A5 expression through the transcription factor ATF4 to compensate for extracellular glutamine acquisition, and silencing ATF4 could relieve resistance [161]. Interestingly, a recent study revealed that the lncRNA SYTL5-OT4 could suppress the autophagic degradation of SLC1A5, which accounts for the resistance of colorectal cancer to regorafenib or bevacizumab, expanding the scope of targeting SLC1A5 to antiangiogenic therapy (AAT) [162]. Furthermore, a review by Marianna et al. provided insights into strategies aimed at enhancing tumor immunity by targeting SLC1A5/SCL3A2 [163].

#### 3.2.2. The GSH Salvage System in Cancer Therapeutic Resistance

The salvage or utilization of GSH, which is crucial for cellular defense against ROS, relies heavily on the activity of key enzymes such as GPX and GST [3,7]. GPXs belong to a multienzyme family whose primary function is to catalyze the reduction of H_2_O_2_ and/or lipid hydroperoxides to water or lipid alcohols while forming disulfide bonds through thiol dehydrogenation to generate GSSG from two molecules of GSH. GPXs consist of eight isozymes (GPX1-8), among which GPX1-4 are the subject of extensive research [123]. We will focus on GPX4 and GPX1 because of their important roles in cancer. While GPX3 is an extracellular lipid peroxidase, GPX4 is the most important intracellular enzyme for detoxifying lipid hydroperoxides, and together with ferroptosis suppressor protein 1 (FSP1), GPX4 and FSP1 constitute two key proteins involved in the defense against ferroptosis [123,164,165]. Recently, emerging evidence has suggested the involvement of drug-tolerant persister (DTP) cells, whose drug-resistant phenotype is temporary and has no resistance mutations, in the failure of antitumor therapy [166]. Studies have indicated that redox signaling is crucial for counteracting the oxidative stress induced by cancer therapies and is essential for sustaining DTP status as well as preventing tumor recurrence [167,168]. One study showed that persister cells of different cancers (breast, melanoma, lung, and ovarian cancers) were vulnerable to GPX4 inhibition, which led to the accumulation of lipid hydroperoxides and ferroptosis upon the withdrawal of ferrostatin-1, a ferroptosis-rescuing antioxidant. When treated with dabrafenib or trametinib, the GPX4-knockout xenograft tumors did not relapse in mice, whereas wild-type tumors did [169]. Another study also validated that resistance to different therapies in cancer depended on a high mesenchymal state, which was determined by the inhibitory effect of GPX4 on ferroptosis [170]. Furthermore, the combined inhibition of GPX4 and mitochondrial dihydroorotate dehydrogenase (DHODH) effectively eradicated GPX_4_^high^ cancer cells, highlighting the synergistic induction of ferroptosis resistance by GPX4 and DHODH [171]. More convincingly, emerging evidence also supported that the loss of GPX4 strengthened the sensitivity of tumor cells to chemotherapy, hormone therapy, radiotherapy, and immunotherapy [172,173,174,175], which, together, reinforced the feasibility of targeting ferroptosis in different cancers [176]. High expression of GPX4 was tightly correlated with increased levels of xCT in breast cancer, showing synergistic regulation within the GSH antioxidant system [177]. The role of GPX1 is to utilize GSH for the reduction of H_2_O_2_ to water, and it also plays a significant role in the management of cancer therapeutic resistance. For instance, GPX1 is involved in cisplatin resistance in ovarian cancer, antitumor immunosuppression in AML, and radioresistance in GBM [178,179,180]. However, silencing GPX1 induced a mesenchymal phenotype and conferred resistance to gemcitabine in PDAC [181], paradoxically suggesting a potential anticancer role of GPX1 in certain cancer types or contexts.

Glutathione S-transferases (GSTs), phase II drug-metabolizing enzymes, also utilize GSH to detoxify exogenous or endogenous electrophilic compounds and hydrophobic GSH conjugates and then exit cells via multiple resistance-associated protein (MRP) transporters [27,122]. The main chemotherapy drugs for ovarian cancer are platinum regimens and paclitaxel derivatives, while GSTs are considered to be the primary enzymes responsible for rendering these drugs ineffective, especially GSTP1, which can be upregulated by G6PD [182]. GSTs can also promote the detoxification of other chemotherapeutic drugs, such as doxorubicin, cyclophosphamide, melphalan, chlorambucil, and irinotecan, by catalyzing conjugating reactions, and different classes of GSTs (with high polymorphism and consisting of different subunits) have varying selectivity toward substrates [183]. The nonenzymatic form of GSTs can also contribute to drug resistance. Given that the MAPK pathways activated by various drugs can activate apoptosis, it has been found that GSTP1 can compete with c-Jun for binding to c-Jun NH2-terminal kinase (JNK) and form a complex, thereby inhibiting c-Jun-mediated apoptosis. GSTP1 can also interact with TNF receptor-associated factor 2 (TRAF2) to attenuate TRAF2-JNK/p38/apoptosis signal-regulating kinase 1 (ASK1)-mediated apoptosis [184]. In HepG2 cells, GSTP1 can directly interact with STAT3 to regulate cell proliferation, but no studies have shown that this interaction is correlated with therapeutic resistance [185]. Given the detailed elucidation of the role of GSTs in cancer drug resistance, this topic will not be reiterated herein [27,186]. These findings suggest that targeting GSTs, along with targeting MRP1 simultaneously, if necessary, may exhibit a synergistic effect in addressing multidrug resistance (MDR) in tumors [24,187].

Since GSH is constantly consumed to combat ROS production, GSSG is reduced to GSH via catalysis by glutathione reductase (GSR), which requires the coenzyme NADPH. Although the role of GSR in tumorigenesis, progression, and therapeutic resistance appears to be less significant than that of GPXs and GSTs, GSR may still pose an obstacle to therapies in certain circumstances. Solute carrier family 27 member 5 (SLC27A5, an enzyme that metabolizes fatty acid and bile acid)-deficient HCC cells were insensitive to sorafenib because GSR was overexpressed in an NRF2-dependent manner, whereas sorafenib combined with the selective GSR inhibitor carmustine (BCNU) strongly promoted ferroptosis, elucidating the suppressive effect of SLC27A5 on GSR [188]. Recently, a compound termed LCS3 was found to specifically hamper the growth of LUAD cells by synergistically inhibiting GSR and TrxR1 [189]. In line with this, the loss of GSR led to the increased reliance of LUAD cells on TrxR, and the inhibition of TrxR with auranofin exhibited synthetic lethality in the presence of GSH deficiency, indicating a reciprocal compensatory effect of GSH/GSR and TRX/TrxR. Hence, targeting TrxR in LUAD and perhaps other cancer patients is a new therapeutic option, given the frequent deletion of chromosome 8p12, where the GSR gene is located, in different cancers [190].

### 3.3. The TRX Antioxidant System in Cancer Therapeutic Resistance

The TRX system comprises thioredoxin (TRX), thioredoxin reductase (TrxR), thioredoxin-interacting protein (TXNIP), and peroxiredoxin (PRDX) [3,191] (Figure 4). Like GPX, PRDX can also reduce H_2_O_2_, peroxynitrite, and organic hydrogen peroxide through reduced TRX, which acts as a cofactor, while TrxR utilizes NADPH to reduce oxidized TRX. The isozymes TRX1/TrxR are located in the cytoplasm and nucleus, and TRX2/TrxR2 are located in the mitochondria. TXNIP is an inhibitor of TRX activity and regulates intracellular redox homeostasis by binding to TRX [192]. Each of these members of the TRX system has been linked to resistance to different therapies in cancer, and these findings are highlighted here.

The protective role of PRDX1/2 against chemotherapy- or radiotherapy-induced oxidative stress has been extensively reported in many studies across various cancer types involving different signaling pathways [193,194,195]. For example, silencing PRDX2 in vitro increased the sensitivity of breast cancer cells to oxaliplatin, and recently discovered natural compounds, ent-kaurane diterpenoids, could also induce apoptosis/ferroptosis in cisplatin-resistant A549 cells by targeting PRDX1/2 [196,197]. Similarly, PRDX1 is important for the functional maintenance of NK cells under oxidative stress, so the overexpression of PRDX1 could greatly strengthen the antitumor immunity of CAR-modified NK cells [198]. PRDX3, which is specific to mitochondria, is stabilized by prohibitin (PHB) in glioma CSCs to suppress the production of mitochondrial ROS, thereby conferring resistance to radiotherapy in glioma [199], and the regulation of stemness by PRDX1/2/3 has also been found in colon cancer resistant to 5-fluorouracil [200,201]. In prostate cancer, increased PRDX4 not only scavenges ROS but also activates AKT/GSK3 signaling to promote cell proliferation in response to radiation therapy [202]. PRDX5 (an atypical 2-cysteine peroxidase) is believed to be closely associated with castration-resistant prostate cancer (CRPC), as it promotes resistance to androgen receptor (AR) inhibitors and gives rise to a DTP state [203]. Interestingly, one study revealed that PRDX6 (an atypical 1-cysteinine peroxidase) has the ability to inhibit ionizing radiation-induced cell senescence and significantly alter the cytokine secretion phenotype, but it is still unclear whether this effect is related to tumor radiosensitivity [204]. PRDXs also facilitate the occurrence of the EMT and modulate immune cell functionality, as described by Yan Liu et al. [205].

In addition to the PRDX family, TRX/TrxRs, which are potent reducing agents, play an indispensable role in maintaining redox balance. They are excessively activated in many cancers and participate in various signal transduction processes to facilitate tumor adaptation to redox remodeling, promote malignant biological behavior, and confer resistance to diverse treatments [191,206,207]. An increasing number of studies have shown that the combination of targeting TRX/TrxR with other therapies shows great promise as an approach. In addition to the synthetic lethality of GSR with TXR/TxrR, as mentioned above, the inhibition of glycolysis could also enhance the reliance of CRC on the TRX1/SLC1A5 pathway [190,208]. The individual use of the glycolysis inhibitor 2-deoxyglucose or the TXR1 inhibitor PX-12 failed to effectively suppress tumor growth in vivo; however, their combination had a significant impact. A genome-wide siRNA library screening also revealed that abrogating TrxR1 simultaneously with the AKT pathway exhibited catastrophic ROS production and synthetic lethality. Therefore, auranofin could solve the problem of NSCLC resistance to the AKT inhibitor MK2206 [209].

A different approach to suppressing the TRX system is to increase the expression of TXNIP. In imatinib-resistant chronic myeloid leukemia (CML), transformed BCR-ABL inhibited *TXNIP* transcription by inducing c-Myc and initiated a glucose-dependent survival program, while the overexpression of TXNIP or the TXNIP agonist JQ1 inhibited the activity of the key enzymes hexokinase 2 (HK2) and lactate dehydrogenase A (LDHA), thereby synergizing with imatinib to kill CML cells and demonstrating the link between TXNIP and metabolism [210]. The development of resistance to doxorubicin presents a significant challenge in treating patients with triple-negative breast cancer (TNBC). However, the c-Myc inhibitor 10058-F4 has been shown to induce TXNIP expression, thereby enhancing ROS-induced DNA damage and effectively diminishing the resistance of TNBC to doxorubicin treatment [211]. Moreover, TXNIP can also be downregulated by miR-301b-3p and miR-27a-3p, which are derived from mesenchymal stem cell (MSC)/M2 macrophage-derived extracellular vesicles and contribute to vincristine/cisplatin-insensitive GC and 5-fluorouracil-resistant HCC, respectively [212,213]. These findings imply that stromal cells have the ability to modulate TXNIP in tumor cells through the vesicular transport of specific miRNAs.

### 3.4. NADPH Antioxidant System in Cancer Therapeutic Resistance

**Figure 5:**
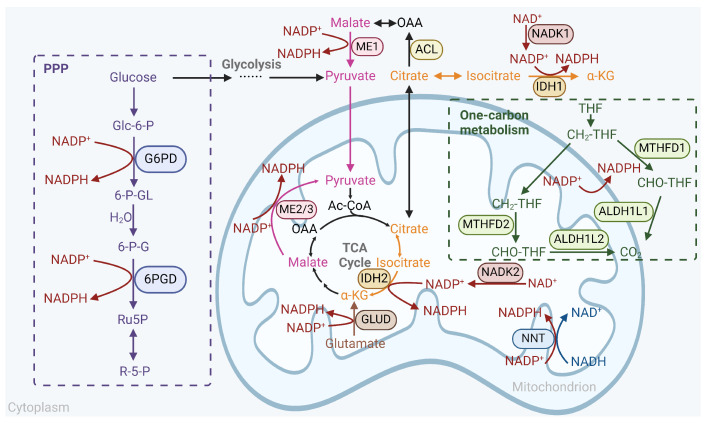
The NADPH antioxidant system in cancer therapeutic resistance. In many cancers, NADPH production is hyperactive to maintain cell redox homeostasis: (1) enhanced PPP flux, characterized by the overexpression of G6PD and 6PGD; (2) the overexpression of IDH and ME; (3) increased glutamate dehydrogenation catalyzed by GLUD; (4) abnormal one-carbon metabolism; (5) the de novo synthesis of NADP+ catalyzed by NADK; (6) the overexpression of NNT utilizing mitochondrial transmembrane proton gradients to generate NADPH.

Reduced NADPH plays a critical role in cellular metabolism, especially for the regeneration of reduced GSH and TRX. The increase in NADPH production can impair numerous cellular physiological processes [3,24]. Not surprisingly, as a means of therapeutic resistance, many NADPH-producing pathways are enhanced in cancers, which we will focus on below [214] (Figure 5).

#### 3.4.1. Pentose Phosphate Pathway

The pentose phosphate pathway (PPP) is a metabolic route for the oxidation and decomposition of glucose involving the enzymatic actions of glucose-6-phosphate dehydrogenase (G6PD) and 6-phosphogluconate dehydrogenase (6PGD), which converts glucose-6-phosphate (Glc-6-P), an intermediate product of glycolysis, into ribulose-5-phosphate (R5P), resulting in the generation of two molecules of NADPH. This pathway is the largest contributor to intracellular NADPH sources and is enhanced in many cancers [214], and increasingly, more studies have shown that the two key enzymes play a critical role in cancer therapeutic resistance.

A metabolic-based CRISPR screening revealed that G6PD played a critical role in the survival of *KRAS*-driven LUAD harboring mutant *KEAP1/NFE2L2*. Its inhibition significantly attenuated TCA metabolism, which was further potentiated by TCA abrogation by the glutaminase inhibitor CB839 [215]. This finding was further validated in *G6PD*-mutant melanoma cells, which exhibited decreased NADPH/GSH levels and increased ROS levels and were sensitized to glutaminase inhibition [216]. In addition to the NRF2-dependent antioxidant program, the overactivation of tumor growth signaling pathways, such as the EGFR/AKT pathway, the mTOR pathway, and the Wnt/β-catenin pathway, also contributes to the overexpression of G6PD, thereby conferring drug resistance, in breast cancer, lung cancer, T-cell acute lymphoblastic leukemia (T-ALL), and multiple myeloma [217,218,219]. Similarly, in PDAC with high G6PD activity, glucose transporter type 1 (GLUT1) is overexpressed to coordinate increased glucose uptake; this process is called “glucomet-PDAC”, which is characterized by a higher chemotherapy resistance rate and worse prognosis [220]. Conversely, targeting G6PD not only sensitized NSCLC to gefitinib but also delayed the acquisition of the EGFR^T790M^ mutation by increasing *EGFR* M790 oxidation and degradation [221]. G6PD inhibition can also promote endoplasmic reticulum stress and the perturbation of autophagic flux, thus synergistically increasing the effect of lapatinib on breast cancer cells [222]. In addition, G6PD can also be upregulated via epigenetic perturbation (histone demethylase gene *KDM5C* depletion), which increases the flux of PPP to enhance NADPH and GSH synthesis to promote ferroptosis resistance in renal cell carcinoma (RCC) [223]. Surprisingly, GSTP1 unexpectedly functions as a novel lactate sensor and noncovalently binds to lactate, thereby augmenting G6PD activity in high-lactate environments to counteract oxidative stress [224]. The above results indicate that G6PD plays a crucial role in NADPH production and is regulated by extremely complex mechanisms. Another enzyme, 6PGD, which catalyzes the oxidative decarboxylation of 6-phosphogluconate (6-PG) to produce a second NADPH, also plays a role in chemotherapy resistance in cancer and can be hyperactivated by many pathways, such as the KEAP1/NRF2/ARE pathway, m6A mRNA methylation, EGFR-mediated phosphorylation, and ME1-mediated allosteric activation [225]. Recently, an autophagy regulatory protein was also found to increase the transcription of 6PGD [226]. Moreover, overactivation of 6PGD is also associated with a poor response to ionizing radiation and immunotherapeutic agents [227,228]. Thus, the direct targeting of the PPP has emerged as a novel therapeutic strategy to overcome therapeutic resistance.

Additionally, alterations in certain extrinsic regulatory factors beyond the PPP can also impact the flux of the PPP, especially glycolytic enzymes such as pyruvate kinase isoform M2 (PKM2), glyceraldehyde-3-phosphate dehydrogenase (GAPDH), fructose-2,6-bisphosphatase 3 (PFKFB3), fructose-2,6-bisphosphatase 4 (PFKFB4), and TP53-induced glycolysis and apoptosis regulator (TIGAR), thereby influencing cellular energy metabolism and redox equilibrium [3,7,214]. For example, TIGAR, a fructose bisphosphatase, has been well established to support the PPP and NADPH production by inhibiting glycolysis, and its knockdown promoted lipid peroxidation and sensitized CRC cells to ferroptosis [229]. Similar observations were noted in radioresistant GBM cells, where the antioxidant capabilities of the PPP were utilized to neutralize the effects of oxidative stress [230,231]. However, considering the intricate functionalities and regulatory networks of these regulators, their involvement in the mechanisms of therapeutic resistance is multifaceted. Further investigation is warranted to ascertain the extent to which they facilitate therapeutic resistance by modulating the PPP and altering the antioxidant capacity of cancer cells.

#### 3.4.2. IDH and ME

The isocitrate dehydrogenase (IDH) family consists of three isoforms: IDH1 is located in the cytosol, and IDH2/3 are located in mitochondria (IDH1/2 are NADP^+^-dependent, and IDH3 is NAD^+^-dependent) [214]. In normal cells, IDH catalyzes the oxidative decarboxylation of isocitrate to α-ketoglutarate (α-KG), and IDH2 is the rate-limiting enzyme of the mitochondrial TCA cycle. However, in *IDH*-mutated tumors, particularly hematological malignancies and gliomas, the oncometabolite 2-hydroxyglutarate (2-HG) is generated from α-KG [232]. This process conversely consumes NADPH and has been reviewed elsewhere [233]. Previous studies on wild-type IDH have shown that receptor tyrosine kinase inhibitors (RTKis) can induce IDH1 expression to support the survival of GBM cells during treatment. However, the genetic or pharmacological inhibition of IDH1 reduced the levels of NADPH and GSH by half and increased the sensitivity of GBM cells to RTKi-induced apoptosis [234]. Coherently, IDH1 silencing imposed metabolic constraints on GBM cells and enhanced their radiosensitization [235]. Compared with IDH^mut^ gliomas, IDH^wt^ primary gliomas tend to exhibit stronger neuronal signaling patterns during relapse, which is correlated with greater aggressiveness and a poorer prognosis [236]. This phenomenon may be partially attributed to enhanced antioxidant capacity. Notably, the United States Food and Drug Administration (FDA)-approved inhibitor of IDH^wt^ (upregulated when glucose was deprived) was effective under low-Mg^2+^ conditions in PDAC [237]. Conversely, the overexpression of IDH2 in TNBC cells reduced the sensitivity to ROS and promoted cell growth [238]. The localization of IDH2 might confer greater significance to the maintenance of mitochondrial homeostasis. Furthermore, IDH also increased lipid and nucleotide biosynthesis through elevated NAPDH levels [239]. Collectively, these results suggest that combination therapies based on IDH inhibition may be an optimal choice for the treatment of cancers, especially glioma [240].

Malic enzymes (MEs) are also located in the cytoplasm (ME1) and mitochondria (ME2/3) and catalyze the oxidative decarboxylation of malate to pyruvate, linking the TCA cycle to glycolysis. Early on, in certain cancer cells, MEs were shown to generate a substantial amount of NADPH comparable to that generated by the PPP [241]. When deprived of sugar, both glycolysis and the PPP were inhibited, leading to the enhanced ability of several cancer cells to upregulate the expression of ME1 for pyruvate synthesis and NADPH production and increased sensitivity to ME1 ablation [242,243]. In pancreatic cancer or gastric cancer, where the *SMAD4* gene is prone to deletion, codeletion of its adjacent gene *ME2* frequently occurs. This increased the dependence of cancer cells on the isozymes M1/M3 [244,245]. Therefore, targeting M1/M3 might effectively eradicate these *SMAD4*-deficient cancers. Furthermore, recent studies have demonstrated the direct role of MEs in drug resistance in NSCLC and AML by regulating cellular NADPH homeostasis and redox metabolism [246,247].

#### 3.4.3. Other Pathways to Generate NADPH

***One-carbon Metabolism.***

The primary role of one-carbon units is to participate in the synthesis of purines and pyrimidines, which are bound to tetrahydrofolate and transported for metabolic processes. Two key enzyme-catalyzed steps generate NADPH, and they are important for maintaining the NADPH/NADP^+^ and GSH/GSSG ratios within cells [124]. Abolishing this pathway sensitizes cells to oxidative stress [248]. One enzyme is methylenetetrahydrofolate dehydrogenase (MTHFD1 in the cytosol and MTHFD2 in the mitochondria), which catalyzes the conversion of 5,10-methylene-tetrahydrofolate (CH2-THF) to 10-formyl-tetrahydrofolate (CHO-THF), and the other is aldehyde dehydrogenase family 1 member L1/L2 (ALDH1L1/2), which catalyzes the conversion of 10-formyl-tetrahydrofolate to tetrahydrofolate and CO_2_. MTHFD is overexpressed in many aggressive cancers and is associated with a poor prognosis [249]. Although its high expression is associated with sensitivity to methotrexate, pemetrexed, and other antifolate drugs, its precise role in therapeutic resistance is not yet clear [250,251]. The relationship between ALDH1L1/2 and cancer is relatively intricate and unexplored, with no direct evidence substantiating its involvement in drug resistance [252].

***De Novo Synthesis.***

NAD^+^ kinases (NADK1 in the cytosol and NADK2 in mitochondria) catalyze the phosphorylation of NAD^+^ to form NADP^+^, which is then reduced to NADPH by various pathways [3]. Studies have shown that NADK-mediated redox adaptation promotes tumorigenesis and metastasis in pancreatic cancer and breast cancer, respectively [253,254]. Oncogenic signals within tumors can also phosphorylate NADK, leading to its hyperactivation. This process subsequently replenishes an increased amount of NADPH, equipping cancer cells with the capacity to overcome a variety of stressors [255,256]. In contrast, the blockade of PI3K/AKT signaling effectively inhibited NADK expression and phosphorylation, leading to a reduction in NADP^+^ synthesis and weakened NADP^+^-stimulated G6PD activity. This ultimately overcame resistance to regorafenib in HCC cell lines [257].

***Dehydrogenation of Glutamate.***

Glutamate dehydrogenases (encoded by the *GLUD1/2* gene) are located primarily in mitochondria and catalyze the reversible oxidative deamination of glutamate to α-KG, utilizing NAD^+^ or NADP^+^ as electron acceptors [214]. GLUDs are closely associated with the TCA cycle and glutamate metabolism, and studies have suggested that they are associated with poor cancer treatment outcomes. One study showed that c-Myc could increase the transcription of the *GLUD* gene, and high levels of GLUD might be associated with the progression of PDAC during therapy [258]. However, the amount of NADPH produced by GLUD probably had little to do with it. In some cancer cells, high GLUD1 expression led to increased α-KG synthesis, which increased the flow of the TCA cycle and accelerated the production of fumarate, which activated the GPX enzyme system and reduced ROS levels. Conversely, GLUD1 deletion disrupted redox balance and impaired tumor growth [259]. The findings of another study, however, presented contradictory outcomes. In NSCLC cells, GLUD1 overexpression promoted the increased entry of glutamate into the TCA cycle, leading to the overload of the electron transport chain (ETC) and the production of significant amounts of ROS, which, in turn, promoted the EMT and resistance to docetaxel [260]. In any case, targeting GLUD may be a new strategy for the treatment of some refractory tumors.

***NNT.***

Nicotinamide nucleotide transhydrogenase (NNT) is located in the mitochondrial inner membrane and utilizes energy from the mitochondrial proton gradient to produce high concentrations of NADPH from NADH under physiological conditions [214]. NNT has been validated to combat oxidative stress in gastric cancer, which promotes tumor progression in all stages and resistance to anoikis [261]. Similar results have been observed in lung cancer and liver cancer [262,263]. A recent study revealed that IL-1β enhanced the acetylation of specific sites on NNT by facilitating the translocation of p300/CBP-associated factor (PCAF) to mitochondria, thereby increasing the affinity between NNT and its substrate NADP^+^. Blocking IL-1β receptors resulted in an imbalance in intracellular iron–sulfur clusters, overcoming immunotherapy resistance and promoting ferroptosis [264]. Additionally, the overexpression of NNT enhanced the sensitivity of lung cancer cells to cisplatin by suppressing protective autophagy [265]. Therefore, NNT may have multiple potential functions, and more research is needed to clarify its relationship with therapeutic resistance.

## 4. Therapeutic Applications for Targeting the Antioxidant Regulation System

**Figure 6:**
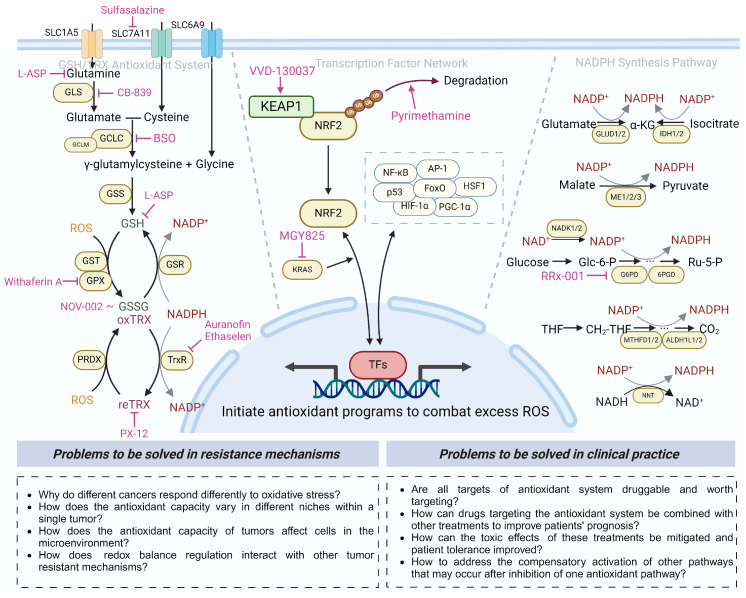
The role of the cancer antioxidant regulation system in therapeutic resistance and scientific questions to be solved. Overall, the cancer antioxidant regulation system consists of three parts: the transcription factor network (regulator), the GSH/TRX antioxidant system (effector), and the NADPH synthesis pathway (facilitator). Among them, the important molecules that play a role in therapeutic resistance are shown in the figure. Drugs that are currently in clinical trials are also shown.

To counteract higher levels of endogenous ROS, cancer cells have evolved adaptive mechanisms that increase their antioxidant properties to exploit the oncogenic effects of high levels of ROS while minimizing oxidative damage. Current treatment methods, such as chemotherapy, radiotherapy, targeted therapy, and immunotherapy, at least partially achieve their therapeutic effects by perturbing the cellular redox status [20]. However, the presence of a vast reservoir of antioxidants within cells poses a challenge in the therapy of various types of cancer due to their ability to neutralize disruptions, as explained above. Thus, targeting the vulnerability of cancer cells that heavily rely on their intrinsic antioxidant systems has emerged as a novel strategy to selectively eliminate them while minimizing cytotoxicity to normal cells. In this section, we summarize the inhibitors of antioxidant systems that are currently undergoing clinical trials or have been officially approved, as well as their mechanisms of action, indications, and current status [266] (Table 2) (Figure 6).

The KEAP1-NRF2-ARE axis, as the central link in cellular antioxidant modulation, may largely lead to poor therapeutic effects, especially in lung cancer. However, there has been a dearth of drugs that specifically target NRF2/KEAP1 in both in vitro studies and clinical applications. NRF2 is generally considered undruggable due to the absence of allosteric pockets [285], so repurposing existing drugs has become a promising way to overcome this bottleneck. A recent high-throughput screening identified the antimalarial drug pyrimethamine as a potent NRF2 inhibitor [267]. Clinical trials of pyrimethamine are planned for locally advanced HNSCC. As oncogenic proteins activate NRF2, targeting KRAS may concomitantly inhibit NRF2, thereby retarding the development of drug resistance [70]. MGY-825, a KRAS inhibitor, is currently undergoing clinical trials in metastatic or unresectable NSCLC harboring *NFE2L2/KEAP1/CUL3* mutations, with the expectation of “killing two birds with one stone”. Additionally, the first KEAP1 activator, VVD-130037, was recently tested in a phase I clinical trial to assess its preliminary efficacy in patients with advanced solid tumors, providing a precise solution for overcoming therapeutic resistance. Despite numerous studies showing that some small molecules, natural compounds, and multitargeted kinase inhibitors can effectively target KEAP1/NRF2 signaling and exhibit varying degrees of antitumor activity in preclinical studies, the clinical translation remains disappointing [9,23]. It should be noted that KEAP1/NRF2 is present in nearly all cells of the body and is involved in numerous vital metabolic pathways. The severe toxicities associated with its targeting may pose a challenge to this therapeutic strategy.

Manipulating the GSH/TRX system has also become an option for cancer treatment, and attempts have been made in clinical practice to target this antioxidant “effector” in combination with other treatment methods. The GSSG mimic NOV-002 was reported to improve response rates in patients with advanced HER-2-negative breast cancer when it was used in combination with doxorubicin and cyclophosphamide [268]. In another phase II study, NOV-002 also extended progression-free survival (PFS) in patients with carboplatin-resistant ovarian cancer [269], but no phase III clinical trials have been conducted in recent years. L-asparaginase (L-ASP) is an FDA-approved chemical agent used to treat acute lymphoblastic leukemia (ALL) that works in part by hydrolyzing glutamine and ablating GSH [22,286]. In a phase II study, eryaspase (erythrocyte-encapsulated asparaginase), in combination with standard chemotherapy, improved OS and PFS in patients with advanced pancreatic cancer [270,271]. However, in a randomized multicenter phase III trial, eryaspase did not achieve the primary objective of improving OS, although it showed a tendency toward a potential benefit, which is worth studying in more cancers. In terms of the specific GSH synthesis and salvage process, although various targeted compounds have been utilized in research [9], the number of compounds that have progressed to clinical trials remains relatively limited. Buthionine sulfoximine (BSO) is a potent inhibitor of GCLC that is generally used in combination with chemotherapy to improve drug resistance [287]. However, its high cytotoxicity to normal cells limits its clinical application. It has shown good tolerability and preliminary efficacy only in pediatric neuroblastoma patients thus far when combined with melphalan [272]. The glutaminase inhibitor telaglenastat (CB-839) plus everolimus was shown to improve PFS in patients with metastatic RCC in a phase II study; however, disappointing results were obtained when this agent was combined with cabozantinib [273,274]. Another study of telaglenastat in combination with nivolumab in participants with RCC, melanoma, and NSCLC was terminated prematurely due to a lack of efficacy (NCT02771626). Sulfasalazine is a sulfonamide antibiotic widely used in inflammatory diseases such as ulcerative colitis and Crohn’s disease. It has recently been repurposed for use as monotherapy or in combination with other therapies for AML, GBM, and CRC by effectively inhibiting xCT [136]. The natural compound withaferin A, a GPX4 inhibitor that induces ferroptosis in vitro [24,288], is also undergoing early-stage clinical trials. Regrettably, there have been no clinical advancements in other drugs that target the utilization of GSH. For the TRX/TrxR system, although the TRX1 inhibitor PX-12 exhibited promising results in in vitro studies and phase I trials, it failed to achieve the anticipated antitumor efficacy in a phase II trial [191,275,276]. In addition, the antirheumatic drug auranofin and the selective TrxR inhibitor ethaselen are currently in early-stage trials and lack sufficient evidence-based medicine results [277]. Therefore, further research is needed to develop preferable treatment combination strategies that can improve the efficacy while minimizing toxicity to the human body.

As G6PD is the most critical enzyme in intracellular NADPH generation, the targeted inhibition of G6PD overexpression in tumor cells is an important strategy. RRx-001, an NLR family pyrin domain-containing 3 (NLRP3) inflammasome inhibitor and NO releaser, was found to inhibit G6PD in different cancer cells [289,290]. It has been demonstrated in early clinical trials to have favorable safety and efficacy when used in combination with other therapies for the treatment of various types of cancer. A phase III trial is currently underway to evaluate its efficacy in the treatment of advanced SCLC combined with platinum regimens [278,279,280,281,282,283,284]. However, due to the diversity of its mechanisms of action, the extent to which it inhibits NADPH synthesis is not clear. Additionally, although there are drugs targeting mutant IDH, no specific inhibitors for the vast majority of tumors with wild-type IDH have been developed [214,237]. Given that NADPH serves as an auxiliary antioxidant, pharmaceutical interventions targeting its biosynthesis for the treatment of cancer are lacking.

## 5. Conclusions and Future Perspectives

In their origin and progression, cancer cells have evolved a more complex antioxidant regulatory system to adapt to stronger oxidative stress, keeping the cell in redox balance on a high platform. This phenomenon can be called “redox reprogramming/resetting”. In general, this regulatory system allows high levels of ROS to become an accomplice to cancers while at the same time attenuating or counteracting their toxic effects on cancer cells. This has also become an important component of the mechanisms by which cancers resist different therapies, as current therapies are generally based on the generation of oxidative stress. Specifically, the intrinsic antioxidant system can be categorized into a transcription factor regulatory system, a GSH/TRX effect system, and an auxiliary NADPH production system. Current theories suggest that the regulatory system is centered around the KEAP1-NRF2-ARE axis and collaborates with other transcription factors to form a hierarchical antioxidant network that responds to oxidative stress. The downstream effector system is precisely regulated by antioxidant programs to coordinate the synthesis, utilization, and regeneration of reduced GSH/TRX and many other GSH/TRX-independent antioxidant factors, such as SODs, catalases, transition-metal-ion-binding proteins, HOMX-1, and NQO1, thereby enhancing cancer therapeutic resistance. In addition, numerous NADPH production pathways, such as the PPP, IDH, ME, one-carbon metabolism, NADK, and GLUT, continuously provide reducing equivalents for the regeneration of GSH/TRX.

Owing to the crucial roles of antioxidant systems in cancer initiation, progression, and therapeutic resistance, manipulating oxidative stress has become a novel strategy for overcoming resistance and eradicating refractory cancers. One approach is to produce an excess ROS burst to overwhelm the antioxidant system. On the other hand, targeting ROS scavengers to disrupt redox homeostasis, which seems more difficult in practice, is the focus of this review. Over the past decade, the utilization of antioxidant inhibitors, either alone or in combination with chemotherapy or radiotherapy, has been extensively explored as a strategy to induce cell death by triggering oxidative stress. Encouraging outcomes have been observed from some of these endeavors. However, as we have observed, most studies are still in the early stages, and many still face significant challenges without achieving the anticipated outcomes. The reasons for the failure of clinical translation may be as follows: (1) the drugs have high toxicity to normal tissues and limited tolerability; (2) the compensatory nature within the regulatory network results in an inhibitor being unable to effectively eliminate antioxidant capacity; (3) the main antioxidant components and therapeutic resistance mechanisms differ among different types of cancer; (4) there is heterogeneity in oxidative stress and antioxidant regulation among different niches within the same tumor; (5) the interaction between the tumor microenvironment and antioxidant inhibitors affects therapeutic efficacy; and (6) many molecules are difficult to target specifically. Unfortunately, our present comprehension of all of these issues is inadequate or even poor. For these reasons, we urgently need to carry out relevant research to better elucidate the regulation of antioxidants in cancer. The advancement of our knowledge of these issues will lead to the development of more efficacious anticancer therapies and better well-being for numerous cancer patients. 

## Figures and Tables

**Table 1:** Mechanisms of NRF2 regulation in therapeutic resistance of different types of cancers.

Cancer Types	Therapies	Specific Resistance Mechanisms	Refs.
***Genetic alterations***			
Lung cancer	Cisplatin	Gain-of-function mutations in NFE2L2 or loss-of-function mutations in KEAP1	[52,53,54,55,56]
	Radiotherapy	Depletion of KEAP1 and impairment of ferroptosis	[57,58]
	PD-(L)1 inhibition	*KEAP1* mutation, damaged immune cell infiltration and CD_8_^+^ T-cell immunity	[59,60,61]
	Gefitinib	*KEAP1* mutation and elevated NRF2 levels	[56]
Gallbladder cancer	5-Fluorouracil	*KEAP1* mutation and activation of NRF2 pathways	[62]
Ovarian cancer	Platinum-based drugs		[63]
Prostate cancer	Paclitaxel, cisplatin, etopside, and irradiation		[64]
Melanoma	Cisplatin, dacarbazine		[65]
HNSCC	Cisplatin	Enhanced antioxidant capacity due to gain-of-function mutations in NFE2L2 or loss-of-function mutations in KEAP1	[66,67]
HCC	NA	*NFE2L2* mutations and increased transcriptional activity of NRF2	[68]
ESCC	NA		[69]
***Transcriptional alterations***			
Pancreatic cancer	NA	Activation of oncogenic proteins such as KRAS/ERK/NRF2 pathway	[70,71]
AML	Cytarabine	Overexpression of NRF2 inhibited MSH2, which induced gene instability-dependent resistance	[72]
Glioma	Radiotherapy, temozolomide	Hypermethylation of CpG islands in promoters of *KEAP1* catalyzed by DNMTs, which suppressed transcription of KEAP1 mRNA	[73]
Colorectal cancer	NA		[74]
Lung cancer	NA		[75,76]
Colorectal cancer	NA	Demethylation of NFE2L2 promoter	[77]
Lung cancer	NA	EZH2 deficiency caused demethylation of histone H3K27, which promoted transcription of NRF2	[78]
***Translational alterations***			
HCC	5-Fluorouracil	MiR-144(-3p), a microRNA that degraded NRF2 mRNA effectively, was lower in cancer cells compared with normal cells	[79]
Lung cancer	Cisplatin		[80]
Breast cancer	NA	MiR-200a degraded KEAP1 mRNA and then induced NRF2-dependent gene expression	[81]
ESCC	Cisplatin, 5-fluorouracil	MiR-432 directly targeted KEAP1 transcripts and then stabilized the NRF2 proteins	[82]
Lung cancer	Paclitaxel, cisplatin, pemetrexed	MiR-6077 inhibited CDKN1A-CDK1-mediated cell cycle arrest and targeted KEAP1, facilitating NRF2-SLC7A11/NQO1 antioxidant axis	[83,84]
	Gefitinib	NRF2-miR-196a axis was upregulated to suppress GLTP	[85]
***Posttranslational Modification***			
Renal cell carcinoma	NA	KEAP1 succination due to accumulation of fumarate in fumarate hydratase-deficient cells	[86]
NA	NA	KEAP1 alkylation by the excessive TCA cycle metabolite itaconate	[87]
NA	NA	KEAP1 lactoylation due to accumulation of glycolysis metabolite glyceraldehyde 3-phosphate	[88]
NA	NA	KEAP1 phosphorylation by multiple kinases	[89]
***Protein–protein interaction***			
Lung cancer	Oxidative stress induced by H_2_O_2_	p21 stabilized NRF2 by competing with KEAP1 and binding NRF2; NRF2 also promotes CDKN1A gene transcription	[90,91]
HCC	Sorafenib, erastin	p62 bound to KEAP1 and confined it to autophagosomes, which stabilized NRF2	[92,93]
ESCC	Cisplatin, paclitaxel	DPP3 bound to KEAP1 and freed NRF2	[94,95]
Colon cancer	Doxorubicin	iASPP bound to KEAP1 and interrupted KEAP1/NRF2 interaction	[96]
Breast cancer	NA	BRAC1 interacted with NRF2 and promoted its stability and activation	[97]

NA: not applicable.

**Table 2:** Clinical trials using drugs targeting antioxidant systems for anticancer therapy.

Drug Name	Mechanism of Action	Cancer Types	Current Status	Trial ID/Refs.
***Drugs targeting KEAP1/NRF2 system***				
Pyrimethamine	Inhibitor of NRF2 by promoting NRF2 ubiquitination and degradation	Locally advanced (stage III-IV) HNSCC (single agent)	Phase I, recruiting	NCT05678348 [267]
MGY825	Inhibitor of KRAS	Advanced NSCLC harboring NFE2L2/KEAP1/CUL3 mutations (single agent)	Phase I, recruiting	NCT05275868
VVD-130037 (BAY 3605349)	Activator of KEAP1	Advanced solid tumors without KEAP1 mutations (single agent)	Phase I, recruiting	NCT05954312
***Drugs targeting GSH/TRX system***				
NOV-002	Oxidized glutathione mimic that alters intracellular GSH/GSSG ratio	HER2-negative IIB-IIIC breast cancer (combination with doxorubicin and cyclophosphamide)/ovarian cancer	Phase II, completed	NCT00499122 [268] NCT00345540 [269]
L-asparaginase	Various mechanisms, includ- ing hydrolyzing glutamine and GSH depletion	Locally advanced or metastatic pancreatic cancer (combination with modified FOLFIRINOX chemotherapy)	Phase I, active	NCT04292743
		Advanced or metastatic pancreatic cancer (combination with gemcitabine or FOLFOX)	Phase IIb, completed	NCT02195180 [270,271]
		Advanced or metastatic pancreatic cancer (combination with gemcitabine or irinotecan-based chemotherapy)	Phase III, completed	NCT03665441
Buthionine sulfoximine (BSO)	Irreversible inhibitor of GCLC that inhibits de novo GSH synthesis	High-risk neuroblastoma (combination with melphalan)	Phase I, completed	NCT00005835 [272]
Telaglenastat hydrochloride (CB-839 HCl)	Inhibitor of glutaminase, in- terfering with glutamate-de- pendent cellular metabolism and redox status	Advanced tumors include TNBC, NSCLC, RCC, mesothelioma, CRC, hematological tumors (sin- gle agent or combination with other therapies)	Phase I, recruit- ing, active or com- pleted	NCT02071862
				NCT02071888
				NCT03965845
				NCT03263429
				NCT03831932
				NCT02071927
				NCT03047993
		Advanced or metastatic solid tumors with specific mutations (single agent)	Phase II, active	NCT03872427
		Melanoma, ccRCC, NSCLC (combination with nivolumab)	Phase I/II, terminated	NCT02771626
		Advanced or metastatic RCC (combination with everolimus or cabozantinib)	Phase II, completed	NCT03163667 [273]
				NCT03428217 [274]
Sulfasalazine	Approved as anti-inflam- matory agent, competitive in- hibitor of Cys/Glu trans- porter xCT, inducing lipid peroxidation and ferroptosis	AML (combination with standard-care induction therapy)	Phase I/II, recruiting	NCT05580861
		Recurrent glioblastoma (combination with γ-knife radiosurgery)/glioma (single agent)	Phase I, completed	NCT04205357 NCT01577966
		Metastatic colorectal cancer (single agent)	Phase I, recruiting	NCT06134388
Withaferin A	Inhibitor of GPX4	High-grade relapsed or metastatic osteosarcoma (single agent)	Phase I/II, unknown status	NCT00689195
PX-12	Inhibitor of thioredoxin-1 (TRX-1)	Advanced pancreatic cancer (stage IV) (single agent)	Phase II, terminated	NCT00417287 [275]
***Drugs targeting GSH/TRX system***				
PX-12	Inhibitor of thioredoxin-1 (TRX-1)	Advanced or metastatic solid tumors (single agent)	Phase I, completed	NCT00736372 [276]
Auranofin	Approved as antirheum- atic drug, with activity tar- geting TrxR	Recurrent ovarian cancer (combination with sirolimus)/advanced or recurrent SCLC or NSCLC (combination with sirolimus)	Phase I/II, active	NCT03456700 NCT01737502
		Relapsed or refractory CLL (single agent)/recurrent glioblastoma (9 repurposed drugs combined with temozolomide)	Phase I/II, completed	NCT01419691 NCT02770378 [277]
Ethaselen	Selective inhibitor of TrxR	High-TrxR-expressing advanced NSCLC (single agent)	Phase I, completed	NCT02166242
***Drugs targeting NADPH generation system***				
RRx-001	Novel epigenetic modula- tor that produces NO and ROS under hypoxic condi- tions and inhibits G6PD and NLRP3 activity	Brain metastases (combination with whole-brain radiotherapy)/high-grade glioma (combination with radiotherapy and temozolomide)/advanced cancers	Phase I/II, completed	NCT01359982 [278] NCT02518958 [279] NCT02215512 [280] NCT02871843 [281]
		Metastatic CRC (combination with irinotecan)/platinum-resistant SCLC (combination with platinum and etoposide)	Phase II, completed	NCT02096354 [282] NCT02489903 [283]
		Third-line or beyond SCLC (combination with platinum regimens)	Phase III, active	NCT03699956 [284] NCT05566041

## Data Availability

Not applicable.
